# Crisis and uncertainty in global health

**DOI:** 10.17843/rpmesp.2025.421.14784

**Published:** 2025-03-10

**Authors:** Marcos Cueto

**Affiliations:** 1 Casa de Oswaldo Cruz/Fiocruz, Rio de Janeiro, Brasil. Casa de Oswaldo Cruz/Fiocruz Rio de Janeiro Brasil

The unilateral withdrawal of the United States of America (USA) from the World Health Organization (WHO) implies the suspension of financial transfers to the agency for ninety days, the departure of U.S. officials assigned to the organization, and the unusual call to seek "credible and transparent" U.S. and international partners to develop activities previously undertaken by the United Nations agency. As noted by Brazilian public health expert and lawyer Deisy Ventura ^1^, while the WHO is not perfect, it is an essential institution for global cooperation and, despite its weaknesses, can be reformed. No other organization possesses the capacity or resources—such as a centralized secretariat that gathers and analyzes global epidemiological data, along with health officials and personnel distributed between the Geneva headquarters, its six regional offices—including PAHO—and the several national offices that enable the swift sharing of research and of health innovations.

Noteworthy, the United States joined the World Health Organization (WHO) in 1948, through a Congressional resolution which stated that, in the event of withdrawal from the institution, the country would issue a notification a year in advance and would fulfill its financial obligations—which now seems unlikely to happen. With the withdrawal, the U.S. would lose participation in the discussions which determine which strains of influenza and SARS-CoV-2 should be used for annual vaccines, would receive delayed access to data on viruses threatening global health, and dialogue with more than seventy WHO collaborating centers based in the U.S.—covering areas such as nursing, environmental health, and pharmacology, among others- would be damaged. The decision would also exclude the U.S. from the International Health Regulations (IHR)—a framework dating back to 1851 that aims at harmonizing the response to health emergencies among the participating countries. These regulations include the obligation of countries to report epidemic outbreaks, standardize quarantine protocols, and define the criteria for declaring a pandemic. Furthermore, the U.S. would be sidelined from ongoing discussions about a potential international pandemic treaty designed to facilitate equitable sharing of vaccines and other medical supplies. In fact, discussion about that very treaty may be one of the real reasons behind the U.S. withdrawal. Republican Party representatives have recently accused the WHO of threatening U.S. sovereignty and pharmaceutical patents through a pandemic treaty aimed at ensuring vaccine equity throughout the world. Indeed, they blocked the signing of a preliminary draft of the treaty in 2024.

It is important to remember that traditionally, WHO funding depended on regular contributions from Member States, calculated based on each country’s wealth and population (with the United States contributing significantly more than minor nations, such as the Caribbean Island states). However, during Ronald Reagan’s presidency (1981-1989), this funding model was questioned. Reagan considered that the U.S. funding - over 25% of the total budget- was unfair, as it only held one vote in the World Health Assembly. His position also reflected the broader neoliberal ideology of the time, which promoted the reduction of the role of governmental and intergovernmental institutions. Since the 1990s, regular U.S. contributions began to stagnate or be delayed, with infrequent increases. The WHO transitioned to depend from voluntary donations, directed to specific objectives, which implied that these funds were no longer part of the budget of the organization. These voluntary contributions came from private foundations such as the Gates Foundation, as well as from countries like Japan, Germany, China, and significantly, from the United States itself. Over time, this led to the consolidation of a parallel budget based on conditional subsidies. Currently, approximately 80% of the WHO’s funding comes from this parallel budget. This model has sparked debate over WHO's ability to set strategic priorities and design comprehensive health programs ^2^.

In 2017, the WHO experienced a historic milestone with the election of Tedros Ghebreyesus as its first African Director-General. An advocate of Primary Health Care, his approach contrasted with the U.S. preference for programs focused on specific diseases. For the first time, the WHO Assembly—composed of 194 member states—held a secret ballot, leaving behind the previous system in which 34 members of the Executive Board selected the Director-General through a process with limited transparency. Tedros’s landslide victory —receiving 133 votes compared to 50 for the United Kingdom’s candidate, David Nabarro—evidenced a strong support from the Global South. It is reasonable to assume that, had a director more aligned with neoliberal trends or a European expert been elected, Trump´s administration would have given a second thought before withdrawing from the WHO.

This withdrawal would cut approximately one-fifth of WHO’s expenditures, and its consequences will be serious and deeply concerning for the continuity of international solidarity, universal access to health care and essential medicines, the acknowledgement of health as a human right, and the role of health in the development of the world’s poorest countries ^3^. It is foreseeable that the fulfillment of the United Nations’ Sustainable Development Goals—particularly the aim of achieving good health for the poor by 2030—will be indefinitely delayed. Additionally, holistic initiatives such as the strengthening of comprehensive health systems, the focus on Social Determinants of Health, and the promotion of Primary Health Care risk being sidelined or altogether forgotten. In conclusion, we are now facing a scenario where, besides fragmentation and rivalry, a lack of long-term vision will prevail.

The most aggressive form of totalitarian, ultranationalist neoliberalism can normalize health inequities—both between and within countries—as being natural and inevitable, while promoting the blaming of victims—including the sick, sexual minorities, and impoverished nations—for future health disasters. This model appears to be aimed at consolidating a form of Selective Global Health, based on aid in exchange for geopolitical loyalty and rooted in a “Culture of Survival,” in which interventions will be palliative and auxiliary ^(^^4^.

While it may be difficult for a health historian to suggest a course of action, I consider that a deeper commitment towards the WHO from other industrialized nations that do not share United States’ view is essential—along with support from the private sector, philanthropic organizations, and, above all, emerging economies. Other institutional actors in global health, such as UNAIDS and the Global Fund to Fight AIDS, Tuberculosis and Malaria, should stand in defense of the WHO and not be intimidated by the scenario. Equally crucial is the active participation of developing countries, many of which still do not fully acknowledge the relevance of global health. A clear example of this gap is the fact that very few Latin American countries have established centers for global health studies. These nations could strengthen South-South cooperation networks and increase their negotiation power as a block, facing China´s likely participation in the leadership of global health, while being the world’s leading producer of Active Pharmaceutical Ingredients (APIs) and the largest producer of COVID-19 vaccines for the Global South. However, this aspiration may not come to reality immediately, as China continues to face internal challenges in coordinating international health activities across its various institutions. For instance, its National Health Commission lacks the authority to align the different ministries, including the Ministry of Foreign Affairs and its International Development Cooperation Agency.

It would not be the first time an international health organization operates without the United States participation. Between 1919 and 1939, the Hygiene Organization of the League of Nations operated without the involvement of the U.S., despite having the support from President Woodrow Wilson, who was ultimately unable to persuade Congress to join. In that organization, Latin American countries such as Peru and Brazil played prominent roles. Beyond the implementation of important public health programs, the organization became a key forum for reflection about social medicine. As a matter of fact, the famous formulation of the WHO Constitution preamble emerged from those debates: health is not merely the absence of disease, but a state of complete physical, mental, and social well-being. An ideal that neither the denialism nor the unenlightenment of any government will be able to erase.
