# Supplementary material for: Crisis and uncertainty in global health
Source: Rev Peru Med Exp Salud Publica. 2025 Mar 10;42(1):4–5. [Article in Spanish] doi: 10.17843/rpmesp.2025.421.14784 (PMC12176014; doi:10.17843/rpmesp.2025.421.14784)
Supplement: Translation

# Translation: Crisis e incertidumbre en la Salud Global

**Authors:** Marcos Cueto

**Affiliations:** 1 Casa de Oswaldo Cruz/Fiocruz, Rio de Janeiro, Brasil.

La retirada unilateral de Estados Unidos de América (EEUU) de la Organización Mundial de la Salud (OMS) significa la suspensión de la transferencia de fondos a esta agencia por noventa días, la salida de los funcionarios estadounidenses asignados a la organización y el inusitado llamado a buscar socios estadounidenses e internacionales “creíbles y transparentes” para realizar las actividades previamente realizadas por la agencia de Naciones Unidas. Como señala la sanitarista y abogada brasileña Deisy Ventura ^1^, si bien la OMS no es perfecta, es una institución esencial para la cooperación mundial y aunque tenga problemas, es reformable. Ninguna otra organización tiene la capacidad ni los recursos-como una secretaría que centraliza y analiza la información epidemiológica mundial, así como funcionarios y personal de salud repartidos entre la sede de Ginebra, las seis oficinas regionales -como la OPS- y las muchas oficinas nacionales que permiten compartir rápidamente investigaciones e innovaciones sanitarias.

Es importante notar que los EEUU se unieron a la OMS en 1948 mediante una resolución del Congreso que establecía que, en caso de abandono, debían notificarlo con un año de antelación y cumplir con sus obligaciones financieras durante ese período (algo que parece no se va a cumplir). Con el retiro, los EEUU perderían las discusiones sobre qué cepas del virus de influenza y SARS-CoV-2 deben usarse para las vacunas anuales; accedería tardíamente a datos sobre los virus que amenazan el planeta y se erosionaría el diálogo con más de setenta centros colaboradores de la OMS que funcionan en los EEUU (en áreas como enfermería, salud ambiental y farmacología entre otras). La decisión, además, excluye a los EEUU del Reglamento Sanitario Internacional -las normas que buscan uniformizar las respuestas a las emergencias sanitarias y cuyo origen se remonta a 1851- incluyendo la obligación de un país de informar sobre un brote epidémico, la estandarización de cuarentenas y los criterios para declarar una pandemia. Quedaría de lado, asimismo, de las discusiones en curso sobre un tratado internacional sobre las pandemias que facilitaría compartir vacunas y otros suministros médicos. De hecho, la discusión de ese tratado puede ser una de las verdaderas razones del retiro de los EEUU. Representantes del partido Republicano acusaron recientemente a la OMS de amenazar la soberanía de Estados Unidos y de las patentes de productos farmacéuticos a través de un tratado pandémico que buscaba distribuir las vacunas de manera equitativa en todo el mundo. De hecho, frustraron que se firmase un primer borrador en el 2024.

Es importante recordar que tradicionalmente, la financiación de la OMS dependía de las contribuciones regulares de los Estados miembros, calculadas en función de la riqueza y la población de cada país (los EEUU aportaba significativamente más que naciones más pequeñas, como las islas del Caribe). Sin embargo, durante la presidencia de Ronald Reagan (1981-1989), surgieron cuestionamientos sobre este modelo. Reagan consideraba injusto que su país financiara más del 25% del presupuesto mientras que contaba con un solo voto en la Asamblea Mundial de la Salud. Su postura reflejaba además la ideología neoliberal que promovía la reducción del papel de los organismos gubernamentales e intergubernamentales. Desde los años noventa, las contribuciones regulares de los EEUU comenzaron a estancarse o retrasarse, con aumentos poco frecuentes. La OMS pasó a depender de donaciones voluntarias y dirigidas a objetivos específicos, lo que significaba que estos fondos no formaban parte del presupuesto regular de la organización. Estas donaciones provenían de fundaciones privadas, como la Fundación Gates, de Japón, Alemania y China, y en gran medida, de los EEUU. Así, se consolidó un presupuesto paralelo basado en estos aportes condicionados. En la actualidad, aproximadamente el 80% del financiamiento de la organización proviene del presupuesto paralelo. Este modelo ha generado un debate sobre la capacidad de la OMS para establecer prioridades estratégicas y diseñar programas integrales ^2^.

En 2017, la OMS vivió un momento histórico con la elección de Tedros Ghebreyesus como su primer Director General africano. Defensor de la Atención Primaria de Salud, su enfoque contrastaba con la preferencia de los EEUU por programas focalizados en enfermedades específicas. Además, por primera vez, la Asamblea de la OMS, compuesta por 194 miembros, realizó una votación secreta, dejando atrás el sistema anterior en el que 34 integrantes del Consejo Ejecutivo seleccionaban al director general en un proceso poco transparente. El importante margen de victoria de Tedros (133 votos frente a los 50 de David Nabarro, candidato del Reino Unido) evidenció un sólido respaldo del Sur Global. Es razonable suponer que, de haber sido elegido un director más alineado con las tendencias neoliberales o un sanitarista europeo, la administración Trump habría pensado dos veces antes de retirarse de la OMS.

Esta salida, cortaría aproximadamente un quinto de los gastos de la OMS, y sus consecuencias son graves y preocupantes para la continuidad de la solidaridad internacional, el acceso universal a la salud y a los medicamentos esenciales, la concepción de la salud como un derecho humano y su papel en el desarrollo de los países más pobres ^3^. Es previsible una postergación indefinida de los Objetivos de Desarrollo Sustentable de Naciones Unidas, que quería dar buena salud a los pobres en el 2030, y pretender que se olviden proyectos holísticos como el del fortalecimiento de sistemas integrales de salud, los Determinantes Sociales de le Salud y la Atención Primaria de Salud. Es decir, un panorama donde además de la fragmentación y rivalidad, parece que se va a entronizar una falta de visión del futuro.

La versión más agresiva de un neoliberalismo totalitario y ultranacionalista puede normalizar las inequidades en salud, tanto entre países como dentro de ellos -como una realidad natural e inevitable- y la culpabilización de las víctimas -enfermos, minorías sexuales y países pobres- de futuros desastres sanitarios. Un modelo al parecer orientado a consolidar una Salud Global Selectiva basada en la ayuda a cambio de lealtades geopolíticas y en la noción de la “Cultura de la Supervivencia” donde las intervenciones sean asistenciales y paliativas ^4^.

Aunque es difícil para un historiador de la salud decir qué debe hacerse, considero que sería imprescindible un compromiso más profundo con la OMS por parte de otros gobiernos de países industrializados no alineados con los EEUU, así como el respaldo del sector privado, la filantropía y, sobre todo, de economías emergentes. Otros actores institucionales de la salud global, como ONUSIDA y el Fondo Mundial para la Lucha contra el SIDA, la Tuberculosis y la Malaria, deberían salir en defensa de la OMS y no verse intimidados. Igualmente, es crucial la participación activa de los países en desarrollo, muchos de los cuales aún no reconocen plenamente la importancia de la salud global. Un claro ejemplo de esta ausencia es que muy pocos países latinoamericanos cuentan con un centro de estudios de salud global. Estos países podrían fortalecer sus redes de cooperación Sur-Sur y aumentar su capacidad de negociación como bloque ante la probable incursión de China -el principal productor mundial de Ingredientes Farmacéuticos Activos (API por sus siglas en inglés) y el mayor exportador de vacunas contra la Covid-19 a los países del Sur Global- en el liderazgo de la salud global. Una aspiración que puede no concretarse de inmediato, porque el país asiático tiene problemas para coordinar las actividades sanitarias internacionales de sus distintas instituciones (su Comisión Nacional de Salud no tiene la autoridad para alinear a los ministerios gubernamentales, incluyendo el Ministerio de Relaciones Exteriores y la Agencia de Cooperación Internacional para el Desarrollo).

No sería la primera vez que una organización de salud internacional opera sin la participación de los EEUU. Entre 1919 y 1939, la Organización de Higiene de la Liga de las Naciones funcionó sin la adhesión norteamericana, a pesar del respaldo del presidente Woodrow Wilson, quien no logró convencer al Congreso de sumarse. En aquella organización, países latinoamericanos como el Perú y Brasil tuvieron un rol destacado. Además de la implementación de valiosos programas sanitarios, este organismo se convirtió en un espacio fundamental de reflexión sobre la medicina social. De hecho, de sus debates surgió la célebre formulación del preámbulo de la Constitución de la OMS: la salud no es simplemente la ausencia de enfermedad, sino un estado de completo bienestar físico, social y mental. Un ideal que ni la torpeza ni el negacionismo de un gobierno podrá borrar.
